# Job Control and Employee Innovative Behavior: A Moderated Mediation Model

**DOI:** 10.3389/fpsyg.2022.720654

**Published:** 2022-05-06

**Authors:** Guolong Zhao, Yuxiang Luan, He Ding, Zixiang Zhou

**Affiliations:** ^1^School of Labor and Human Resources, Renmin University of China, Beijing, China; ^2^School of Economics and Management, North China Electric Power University, Beijing, China

**Keywords:** job control, employee innovative behavior, moderated mediation model, mindfulness, creative self-efficacy

## Abstract

The revolution of self-management and organizational democracy is gaining momentum with the development of new technologies. How to stimulate high employee innovation behavior is critical to an organization’s success. In this study, we built and verified a theoretical model to explore the effect of job control (JC) on employee innovative behavior (EIB), the mediating effect of creative self-efficacy (CSE), and the moderating effect of mindfulness (MF), based on the self-determination theory (SDT). For this quantitative study, a 31-item questionnaire was used to collect data from five Internet companies with 329 Chinese employees. AMOS 24.0 software was used to calculate CFA. SPSS26.0 software was used to calculate means, standard deviations, correlations, and regression analysis. The results indicate that a moderated mediation model among JC, CSE, EIB, and MF is supported. Further, JC was positively related to EIB *via* CSE. Moreover, MF moderated the relationship between JC and EIB and the mediating role of CSE.

## Introduction

With the development of the Internet, such as the Internet of Things (IoT), artificial intelligence (AI), and other new technologies, organizations are increasingly facing an uncertain, ambiguous, and complex external competitive environment. Dehumanizing, mechanistic, or countervailing authoritarian leadership is facing many challenges, which urgently prompt organizations to respond quickly and innovate actively. If an organization wants to survive and develop, it must constantly innovate. As a result, it is becoming more and more important for organizations to cultivate, develop, and utilize the innovative potential of employees. Individual innovation behavior is critical to an organization’s success (de Jong and den Hartog, 2010). In recent years, enterprises represented by Internet companies have adjusted their organization structure, gradually formed a flat management mode, and given employees more control over their work to encourage employees to innovate.

To some extent, the revolution of self-management and organizational democracy conform to the management trend of encouraging employee innovation. Employee innovative behavior, defined as “the new ideas and methods in products and processes generated by employees based on existing conditions,” which includes the generation of new ideas and the completion of those new ideas (Scott and Bruce, 1994; Janssen et al., 2004; Stashevsky et al., 2006), is very important for organizational innovation (Su et al., 2019).

Scholars have paid a lot of academic attention to researching the antecedents of EIB, drawing on different theories. SDT (Ryan and Deci, 2000b; Deci and Ryan, 2014; Deci et al., 2017) may be the most widely used theory that explains the antecedents of EIB. SDT argues that when people are intrinsically motivated, they are creative and productive (Deci and Ryan, 2000, 2010; Deci et al., 2017). Drawing on SDT, many constructs could influence EIB, such as intrinsic motivation (Zhang and Bartol, 2010; Yidong and Xinxin, 2013; Su et al., 2020), psychological empowerment (Singh and Sarkar, 2012, 2019; Schermuly et al., 2013), and job autonomy (Dhar, 2016; Giebels et al., 2016; Lee et al., 2021). These constructs are all related to the need for autonomy, which is the core construct of SDT (Deci and Ryan, 2000, 2014; Deci et al., 2017). Job control refers to employees’ perceived ability to exert some control over their work environment to make it more rewarding and less threatening (Bond and Flaxman, 2006), which seems to also affect the need for autonomy.

However, there is no evidence that job control can promote EIB. In addition, mindfulness (Brown and Ryan, 2003) and creative self-efficacy (Malik et al., 2015) may also influence innovative behavior drawing on SDT. This study aimed to examine the mediating roles of CSE and the moderating roles of MF in the relationship between JC and EIB. We built a moderated mediating model concluding JC, MF, CSE, and EIB. To test our model, we used a total of 329 samples from Internet companies in which innovation is very crucial.

The structure of the study refers to the mainstream literature format (Iqbal et al., 2021; Rasool et al., 2021). This article is organized as follows. Section 2 presents the SDT theory and hypotheses development, Section 3 presents the research methods, and Section 4 explains the statistical analysis of this study. Section 5 presents the discussion, and Section 6 explains the concluding remarks. Finally, the final section of this study presents the limitations as well as future research directions.

## Theory and Hypotheses Development

### Self-Determination Theory

Self-determination theory argued that there are three types of basic psychological needs: the need for autonomy, competence, and relatedness (Deci and Ryan, 2000; Ryan and Deci, 2000a; Deci et al., 2017). The need for autonomy refers to the need to be the origin of their behaviors and choices; the need for competence means the need to be competent, effective, and masterful; and the need for relatedness implies the need to experience a sense of meaningful connection with at least some other people (Deci and Ryan, 2000; Ryan and Deci, 2000a; Deci et al., 2017; Sheldon and Prentice, 2019). SDT argued that when basic psychological needs are met, people are likely to be intrinsically motivated (Deci and Ryan, 2010; Deci et al., 2017).

Intrinsic motivation is a type of motivation based on people’s natural interest in various activities that provide novelty and challenge (Deci and Ryan, 2010), which is the most important concept of SDT. When people are intrinsically motivated, they are more creative and productive (Deci and Ryan, 2010; Deci et al., 2017). Drawing on SDT, scholars tried to find different constructs to fulfill the basic psychological needs.

### Job Control and Employee Innovative Behavior

Job control may be a potential predictor of EIB. JC reflects employees’ perceived ability to exert some influence over their work environment (Bond and Flaxman, 2006). JC includes timing control and method control (Jackson et al., 1993; Bond and Flaxman, 2006). Timing control refers to the individual’s ability to determine the scheduling of their work behavior, whereas method control refers to individual choice in how to carry out given tasks (Jackson et al., 1993). If people can control time and choice when they behave, they behave autonomously; they control their own behavior rather than being controlled by the environment. As a result, the need for autonomy is satisfied. According to SDT (Deci and Ryan, 2000; Deci et al., 2017), when the need for autonomy is satisfied, people are intrinsically motivated and in turnbecome more creative and productive. Previous studies have provided evidence that the need for autonomy is positively related to EIB (Battistelli et al., 2013; Orth and Volmer, 2017; Slåtten et al., 2020). Thus, we propose the following hypothesis:

Hypothesis 1: Job control will positively influence employee innovative behavior.

### The Mediating Role of Creative Self-Efficacy

Creative self-efficacy is a specific form of self-efficacy that refers to an individual’s belief in their ability to creatively complete tasks and achieve creative results (Tierney and Farmer, 2002). CSE is a core concept derived from self-efficacy theory (SET) (Sweet et al., 2012), which argues that people do what they do because they believe they can. SDT and SET are well aligned because they are based on the ideology that humans are agents of their actions (Sweet et al., 2012). Interestingly, SDT also has a concept called the need for competence, which means they need to be competent, effective, and masterful. Fulfilling the need for competence also motivates people (Ryan and Deci, 2000b; Sheldon and Prentice, 2019). The degree of freedom of action in the workplace (job control) may affect the degree of availability of resources that support the fulfillment of competence (Karanika-Murray et al., 2017).

Specifically, the need for autonomy may support the need for competence. SDT posits the following causal sequence: autonomy support → changes in perceived competence → changes in intrinsic motivation (Guay et al., 2001). JC will influence perceived competence, the fulfillment of which fosters purpose and self-regulation (Karanika-Murray et al., 2017). According to the definition of CSE (Tierney and Farmer, 2002), CSE reflects perceived competence. In addition, previous studies have shown that CSE is positively related to EIB (Michael et al., 2011; Su et al., 2019). In sum, we argued that CSE mediates the relationship between JC and EIB. Thus, we propose the following hypothesis:

Hypothesis 2: Creative self-efficacy will mediate the positive impact of job control on employee innovative behavior.

### The Moderating Role of Mindfulness

Mindfulness is purposefully and non-judgmentally paying attention to the present moment (Giluk, 2009), which is defined as “keeping one’s consciousness alive to the present reality” (Hanh, 1976). Brown and Ryan (2003) argued that this mindful capacity varies within individuals because it can be sharpened or dulled by a variety of factors. Being mindful means paying close attention to and being aware of what is happening in the present moment (Schultz et al., 2014). Ryan and Deci (2017) argued that MF facilitates greater autonomy and integrated self-regulation. When the need for autonomy is satisfied, people become intrinsically motivated. People with a high level of mindfulness would like to fulfill the need for autonomy because they feel that they can control their behaviors and can be self-determined at all times. More specifically, we argued that people with high levels of mindfulness would strengthen the relationship between JC and EIB. A survey by Schultz et al. (2014) provided evidence that MF moderated the relation between work climate and psychological need satisfaction. Thus, we propose the following hypothesis:

Hypothesis 3: MF will moderate the influence of JC on EIB, such that the influence will be more positive when an employee has a high level of MF and is less positive when an employee has a low level of MF.

**FIGURE 1:**
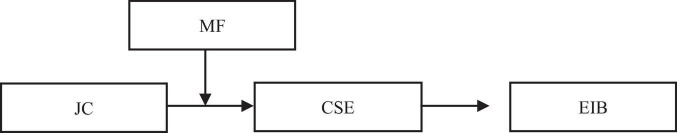
The proposed conceptual model. JC, job control, MF, mindfulness, CSE, creative self-efficacy, EIB, employee innovative behavior.

Drawing on hypothesis 2 and hypothesis 3, we expect that MF could also moderate the mediating effect of CSE in the relationship between JC and EIB. Specifically, the indirect influence of CSE on JC and EIB will be stronger when MF is higher. Taken together, we put a conceptual model (see Figure 1).

## Research Methods

A quantitative research approach was used in this study. The online survey method (Enterprise WeChat) was used for data collection. The reasons for online data collection were as follows: first, during the COVID-19, there was an increase in the use of an online office in the IT industry (Rasool et al., 2021); second, it is a low-cost method of data collection; and third, it is very convenient and comfortable for internet employees to answer online surveys. Hence, an online survey is ideal for data collection in this study. The study was cross-sectional in nature and was based on a convenience sample. For survey analysis, authors must first design the research instrument to collect the data (Rasool et al., 2019, 2020).

### Instrument Development

In this study, we designed a questionnaire for data collection, and the constructed hypotheses served as the foundation (Rasool et al., 2019). The questionnaire of key variables comprised 26 items scored with a five-point Likert scale (1 = strongly disagree to 5 = strongly agree). Prior to the final data collection, the questionnaire’s reliability and validity were checked by two academic professors, five Ph.D. students, and 10 professionals, all of whom had sufficient knowledge of the research objectives. Finally, some recommended changes were made to modify the instrument to meet the objectives.

### Data Collection and Sampling

Data were collected from employees working in five Internet companies in China’s capital city of Beijing. Before data collection, we informed respondents that the confidentiality of their responses was assured and that the information collected would only be used for research purposes. Furthermore, through Enterprise WeChat and emails, we distributed 400 questionnaires among senior managers, middle-level managers, and administrative staff and received a total of 329 useable responses.

### Variables and Measures

Since the original scales were written in English, we invited two Ph.D. candidates to translate all items into Chinese and then back into English following the commonly used back-translation procedure (Su et al., 2019).

### Control Variables

In this study, we controlled for age, gender, tenure (working experience), education level, and position level. Age, tenure, position level, and education level may influence the human capital of employees (Binnewies et al., 2008), which might, in turn, influence employee innovative behavior.

### Job Control

Job control was measured using a five-item scale developed by Jackson et al. (1993). An example sample item is “Do you decide on the order in which you do things?” This variable was rated on a five-point Likert scale (from 1 = almost never to 5 = almost always). The Cronbach’s alpha for this measure was 0.919. The items used in the study were considered valid because their alpha values were above the standard value of 0.70 and higher (Rasool et al., 2021). So, the items we used in this research instrument are valid.

### Mindfulness

Mindfulness was measured using a six-item scale developed by Brown and Ryan (2003). An example sample item is “I could be experiencing some emotion and not be conscious of it until sometime later.” This variable was rated using a five-point Likert scale (from 1 = almost never to 5 = almost always). The Cronbach’s alpha for this measure was 0.941. The items used in the study were considered valid because of their alpha value above the standard value of 0.70 and higher (Rasool et al., 2021). So, the items we used in this research instrument are valid.

### Creative Self-Efficacy

Creative self-efficacy was measured using an eight-item scale developed by Carmeli and Schaubroeck (2007). An example sample item is “I will be able to achieve most of the goals that I have set for myself in a creative way.” This variable was rated using a five-point Likert scale (from 1 = not at all to 5 = to a large extent). The Cronbach’s alpha for this measure was 0.952. The items used in the study were considered valid because their alpha values were above the standard value of 0.70 and higher (Rasool et al., 2021). So, the items we used in this research instrument are valid.

### Employee Innovative Behavior

Employee innovative behavior was measured using a six-item scale developed by Scott and Bruce (1994). An example sample item is “I would search out new working methods, techniques, or ideas in daily work.” This variable was rated using a five-point Likert scale (from 1 = strongly disagree to 5 = strongly agree). The Cronbach’s alpha for this measure was 0.806. The items used in the study were considered valid because their alpha value was above the standard value of 0.70 and higher (Rasool et al., 2021). So, the items we used in this research instrument are valid.

### Respondents’ Summary

Of the 400 questionnaires distributed, 354 completed questionnaires were returned, resulting in a response rate of 88.5%. Of this, 25 were not useable, and only 329 questionnaires were included for further analysis. In this study, we used descriptive statistics. The proportion of respondents under the age of 30 was 59.27%, of those between the ages of 30 and 40 was 36.78%, and of those above the age of 40 was 3.95%. Men made up approximately 51.37% of respondents in this study, while women made up approximately 48.63%. Similarly, we collected data from senior managers (5.17%), middle-level managers (18.54%), administrative employees (21.28%), and staff (55.01%). Finally, the respondents’ education levels included post-graduate (41.03%), undergraduate (55.32%), and others (3.65%). The detailed sample demographics are presented in Table 1.

**TABLE 1:** Respondents’ summary.

Characteristics	Category	Frequency (n)	Percentage (%)
Age	Under 30	195	59.27%
	30–40	121	36.78%
	Above 40	13	3.95%
Gender	Male	169	51.37%
	Female	160	48.63%
Tenure	Under 3 years	171	51.97%
	3–10 years	139	42.25%
	Above 10 years	19	5.78%
Position	Senior manager	17	5.17%
	Middle manager	61	18.54%
	Administrative staff	70	21.28%
	Staff	181	55.01%
Education	Post-graduate	135	41.03%
	Undergraduate	182	55.32%
	Others	12	3.65%
			

## Analysis and Results

### Confirmatory Factor Analysis

To check whether JC, MF, CSE, and EIB could be mutually discriminated, we used AMOS 24.0 to conduct the CFA (see Table 2). The four factors model was compared against three-factor, two-factor, and single-factor models. We found that the four-factor model fit the data better than the three other measurement models (χ^2^/df = 781.23/293 = 2.67 < 3, RMSEA = 0.071 > 0.050, CFI = 0.932 > 0.900, TLI = 0.924 > 0.900, SRMR = 0.048 < 0.050).

**TABLE 2:** Results of CFAs.

Model	χ^2^	*df*	χ^2^/*df*	RMSEA	CFI	TLI	SRMR
One-factor model(JC + MF + CSE + EIB)	3317.66	299	11.09	0.175	0.579	0.542	0.120
Two-factor model(JC + MF, CSE + EIB)	2233.48	298	7.49	0.141	0.730	0.705	0.149
Three-factor model(JC, MF, CSE + EIB)	1204.94	296	4.07	0.097	0.0873	0.861	0.088
Four-factor model(JC, MF, CSE, EIB)	781.23	293	2.67	0.071	0.932	0.924	0.048

### Common Method Variance

Since we used self-reported data, the common method variance may exist (Chang et al., 2010). Harman’s single-factor test was used to check potential common method variance. The first single factor explained 30.25% (<40%) of the variance, demonstrating that the common method variance of this study did not pose a serious threat to our results.

### Descriptive Statistical Analysis

We calculated the means, standard deviations, and correlations between JC, MF, CSE, and EIB (see Table 3). JC is positively related to CSE (*r* = 0.514, *p* < 0.01) and EIB (*r* = 0.677, *p* < 0.01). These results offer preliminary evidence for our hypotheses.

**TABLE 3:** Descriptive analysis and correlations among main variables.

Variable	Mean	SD	JC	MF	CSE	EIB
JC	3.830	0.876	1	0.582**	0.514**	0.677**
MF	3.555	0.909	0.582**	1	0.441**	0.601**
CSE	3.347	0.947	0.514**	0.441**	1	0.577**
EIB	3.608	0.792	0.677**	0.601**	0.577**	1

N = 329; **p < 0.01.

### Hypothesis Testing

We used hierarchical regressions to test our hypotheses (see Table 4). For Hypothesis 1, comparing Model 4 and Model 5, we found that JC is significantly related to EIB (β = 0.680, *p* < 0.001) after controlling for five control variables. Thus, Hypothesis 1 was supported.

**TABLE 4:** Hierarchical regressions for main study variables.

Variable	CSE			EIB		
	Model 1	Model 2	Model 3	Model 4	Model 5	Model 6
Gender	0.031	0.051	0.033	0.006	0.032	−0.006
Age	0.018	−0.011	0.011	0.012	−0.027	0.018
Position level	0.074	0.052	0.004	0.045	0.016	−0.028
Education level	0.027	0.003	0.012	−0.021	−0.053	−0.040
Tenure	0.070	0.079	0.110*	−0.008	0.005	0.019
JC		0.513***	0.449***		0.680***	0.491***
CSE						0.321***
MF			0.248***			
JC × MF			0.172***			
R^2^	0.016	0.277	0.328	0.003	0.462	0.525
Adjusted R^2^	0.001	0.263	0.311	−0.012	0.452	0.515
F	1.064	20.551***	19.536***	0.215	46.102***	50.75***

N = 329; *p < 0.05 and ***p < 0.001.

For Hypothesis 2, Models 1, 2, 4, and 6 demonstrated that CSE mediated the relationship between JC and EIB [JC positively influenced CSE (β = 0.513, *p* < 0.001) and CSE positively influenced EIB (β = 0.321, *p* < 0.001)]. Furthermore, we used the Bootstrap method (Hayes, 2017) to test the indirect effect of CSE on JC and EIB. The result showed 95% CI = [0.095,0.231] that does not include “zero.” Thus, Hypothesis 2 was supported.

**FIGURE 2:**
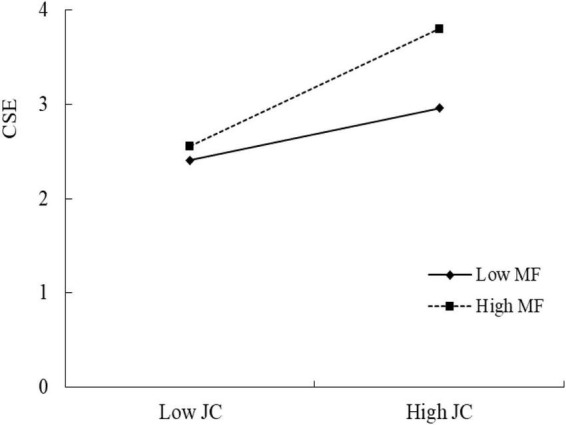
The moderating effect of mindfulness (MF) on the influence of job control (JC) on creative self-efficacy (CSE).

For Hypothesis 3, comparing Model 1, Model 2, and Model 3, JCxMF is significant (β = 0.172, *p* < 0.001). Thus, Hypothesis 3 was a moderated mediation model. From Figure 2, we can clearly see the moderating effect of MF.

We also tested the moderated mediation model by following the guidance of Hayes (2017). The results are shown in Table 5. We found that index = 0.033 and 95%CI = [0.006,0.074] did not include “zero,” which provided evidence for the moderated mediation model. More specifically, we found that the indirect effects increase when MF goes from low to high (β = 0.098 to β = 0.152, 95% CI does not include “zero”). Thus, the moderated mediation model supported the hypothesis.

**TABLE 5:** Results of the moderated mediation model.

Variable	CSE			
	Indirect effect	SE	LLCI	ULCI
Low MF	0.098	0.028	0.041	0.155
Medium MF	0.131	0.031	0.073	0.193
High MF	0.152	0.037	0.084	0.230

## Discussion

We tested the relationship between JC and EIB, as well as the mediating role of CSE and the moderating role of MF. We collected 329 valid data from Internet companies to test our hypotheses. The results supported our hypotheses, and we planned to discuss our contributions based on our findings.

First, we found that JC was positively associated with EIB. To the best of our knowledge, our study is one of the first to show the impact of JC on the EIB domain. A survey study by Martín-Hernández et al. (2020) showed that JC moderated the relationship between demand and EIB. Our study differed from that of Martín-Hernández et al. (2020), in that our study was based on the SDT perspective, while their study was based on the job demand-control perspective. JC is important to employees because JC can fulfill their basic psychologic needs, hence facilitating EIB.

Second, CSE mediated the relationship between JC and EIB. By showing how JC indirectly affects EIB *via* MC, our study contributes to a better understanding of the consequences of JC. Previous studies showed that CSE was positively related to EIB (Michael et al., 2011; Ding and Quan, 2021; Ji and Yoon, 2021). It was clear that CSE could predict EIB because these people who are competent in doing certain things would like to achieve them according to SET (Sweet et al., 2012). Nonetheless, we based our hypotheses on SDT. Scholars tried to integrate SET and SDT into a model, which showed that autonomy influenced confidence (Sweet et al., 2012, 2014). Confidence is similar to the need for competence according to SDT (Ryan and Deci, 2000b; Deci et al., 2017) and is also similar to self-efficacy according to SET. The need for autonomy, which is the core concept of SDT, may bolster the need for competence and the need for relatedness (Deci and Ryan, 2014; Ryan and Deci, 2020). Numerous studies support SET and SDT. We argued that CSE or self-efficacy might be an important construct to integrate SDT and SET. Our study provides evidence showing the need to integrate SET and SDT.

Third, MF moderated the relationship between JC and CSE. Ryan and Deci (2017) argued that MF promotes greater autonomy and integrated self-regulation. Recently, a meta-analysis by Donald et al. (2020) showed that MF was positively related to intrinsic motivation, which is influenced by autonomy. MF is a state of people that would influence their motivation. In our studies, people with a high MF level would be more likely to exert control over their jobs and become more self-determined, which resulted in increased self-efficacy. This is the first study to evaluate the relationship between JC, MF, and CSE from an SDT perspective. Consistent with a previous survey study by Montani et al. (2020), which showed MF moderated the relationship between workload and EIB, our studies revealed that MF had a moderating effect. Our findings may shed light on an important boundary condition that strengthens the relationship between JC and CSE.

### Practical Implications

The current study provided managers and organizations with relevant and meaningful guidance. To begin with, people who are working in Internet companies have to carry put tasks with innovative behavior every day due to the rapid evolution of technologies and the market. Our studies show that JC could influence EIB. Managers should create an environment in which their followers feel empowered to control their work. Fortunately, many managers in Internet companies encourage their employees to exercise control over their work, which, in turn, has influenced EIB. Moreover, our results indicate that CSE mediates the relationship between JC and EIB, so increasing employee CSE is important. Managers should provide timely feedback to their subordinates. Such feedback, which conveys information about employees’ ability to accomplish the job well, could influence the need for competence and improve the CSE. Last but not least, MF plays a significant role in EIB. Brown and Ryan (2003) argued that MF could be improved by effective training. Indeed, employees got so much information every day that it may be deeply confusing. MF could help employees to concentrate on themselves and become more self-determined. If an organization can develop employees’ MF *via* suitable training, employees may become more creative, productive, and healthy.

## Conclusion

The current study indicates that job control has a positive influence on employee innovative behavior. Specifically, we found that creative self-efficacy could mediate the relationship between job control and employee innovative behavior. Moreover, our results show that mindfulness of employees not only moderates the direct influence of job control on employees’ creative self-efficacy but also moderates the mediating role of employee creative self-efficacy in the relationship between job control and employee innovative behavior.

## Limitations and Future Directions

This study is not without its limitations. First, we used cross-section data to test our hypothesis. Cross-sectional data may have low statistical power for inferring causal relationships. We encourage future studies to collect data from a variety of resources and from different times (Ding and Yu, 2020). Second, we tested our hypotheses using a sample of employees from Internet companies in China, which might limit the cross-company applicability because employees from other industries, such as manufacturing, could not control their job as flexibly as employees from Internet companies. Future research should take samples from companies that come from different industries.

## Data Availability Statement

The raw data supporting the conclusions of this article will be made available by the authors, without undue reservation.

## Author Contributions

GZ put the idea and wrote the introduction and theory and hypotheses. HD and YL wrote the method and discussion. HD and ZZ collected the data. All authors contributed to the article and approved the submitted version.

## Conflict of Interest

The authors declare that the research was conducted in the absence of any commercial or financial relationships that could be construed as a potential conflict of interest.

## Publisher’s Note

All claims expressed in this article are solely those of the authors and do not necessarily represent those of their affiliated organizations, or those of the publisher, the editors and the reviewers. Any product that may be evaluated in this article, or claim that may be made by its manufacturer, is not guaranteed or endorsed by the publisher.

## References

[B1] Battistelli A. Montani F. Odoardi C. (2013). The impact of feedback from job and task autonomy in the relationship between dispositional resistance to change and innovative work behaviour. *Eur. J. Work Organ. Psychol.* 22 26–41. 10.1080/1359432x.2011.616653

[B2] Binnewies C. Ohly S. Niessen C. (2008). Age and creativity at work: the interplay between job resources, age and idea creativity. *J. Manag. Psychol.* 23 438–457. 10.1108/02683940810869042

[B3] Bond F. W. Flaxman P. E. (2006). The ability of psychological flexibility and job control to predict learning, job performance, and mental health. *J. Organ. Behav. Manage.* 26 113–130. 10.1186/s12913-016-1423-5 27409075 PMC4943498

[B4] Brown K. W. Ryan R. M. (2003). The benefits of being present: mindfulness and its role in psychological well-being. *J. Pers. Soc. Psychol.* 84 822–848. 10.1037/0022-3514.84.4.822 12703651

[B5] Carmeli A. Schaubroeck J. (2007). The influence of leaders’ and other referents’ normative expectations on individual involvement in creative work. *Leadersh. Q.* 18 35–48. 10.1016/j.leaqua.2006.11.001

[B6] Chang S.-J. Van Witteloostuijn A. Eden L. (2010). From the editors: common method variance in international business research. *J. Int. Bus. Stud.* 41 178–184. 10.1057/jibs.2009.88

[B7] de Jong J. den Hartog D. (2010). Measuring innovative work behaviour. *Creat. Innov. Manage.* 19 23–36. 10.1111/j.1467-8691.2010.00547.x

[B8] Deci E. L. Ryan R. M. (2000). The “what” and “why” of goal pursuits: human needs and the self-determination of behavior. *Psychol. Inquiry* 11 227–268. 10.1207/s15327965pli1104_01

[B9] Deci E. L. Ryan R. M. (2010). Intrinsic motivation. *Corsini Encycl. Psychol.* 1–2. 10.1002/9780470479216.corpsy0467

[B10] Deci E. L. Ryan R. M. (2014). “Autonomy and need satisfaction in close relationships: relationships motivation theory,” in *Human Motivation and Interpersonal Relationships*, ed. Weinstein N. (Dordrecht: Springer), 53–73. 10.1007/978-94-017-8542-6_3

[B11] Deci E. L. Olafsen A. H. Ryan R. M. (2017). Self-determination theory in work organizations: the state of a science. *Annu. Rev. Organ. Psychol. Organ. Behav.* 4 19–43. 10.1146/annurev-orgpsych-032516-113108

[B12] Dhar R. L. (2016). Ethical leadership and its impact on service innovative behavior: the role of LMX and job autonomy. *Tour. Manage.* 57 139–148. 10.1016/j.tourman.2016.05.011

[B13] Ding H. Quan G. (2021). How and when does follower’s strengths-based leadership relate to follower innovative behavior: the roles of self-efficacy and emotional exhaustion. *J. Creat. Behav.* 55 591–603. 10.1002/jocb.473

[B14] Ding H. Yu E. (2020). Follower strengths-based leadership and follower innovative behavior: the roles of core self-evaluations and psychological well-being. *Rev. Psicol. Trabajo Organ.* 36 103–110. 10.5093/jwop2020a8

[B15] Donald J. N. Bradshaw E. L. Ryan R. M. Basarkod G. Ciarrochi J. Duineveld J. J. (2020). Mindfulness and its association with varied types of motivation: a systematic review and meta-analysis using self-determination theory. *Pers. Soc. Psychol. Bull.* 46 1121–1138. 10.1177/0146167219896136 31884892

[B16] Giebels E. de Reuver R. S. Rispens S. Ufkes E. G. (2016). The critical roles of task conflict and job autonomy in the relationship between proactive personalities and innovative employee behavior. *J. Appl. Behav. Sci.* 52 320–341. 10.1177/0021886316648774 27536008 PMC4971615

[B17] Giluk T. L. (2009). Mindfulness, big five personality, and affect: a meta-analysis. *Pers. Individ. Diff.* 47 805–811. 10.1016/j.paid.2009.06.026

[B18] Guay F. Boggiano A. K. Vallerand R. J. (2001). Autonomy support, intrinsic motivation, and perceived competence: conceptual and empirical linkages. *Pers. Soc. Psychol. Bull.* 27 643–650. 10.1177/0146167201276001

[B19] Hanh T. N. (1976). *Miracle of Mindfulness.* Boston: Beacon.

[B20] Hayes A. F. (2017). *Introduction to Mediation, Moderation, and Conditional Process Analysis: A Regression-Based Approach.* New York, NY: Guilford publications.

[B21] Iqbal J. Qureshi N. Ashraf M. A. Rasool S. F. Asghar M. Z. (2021). The effect of emotional intelligence and academic social networking sites on academic performance during the COVID-19 pandemic. *Psychol. Res. Behav. Manage.* 14 905–920. 10.2147/PRBM.S316664 34234587 PMC8254613

[B22] Jackson P. R. Wall T. D. Martin R. Davids K. (1993). New measures of job control, cognitive demand, and production responsibility. *J. Appl. Psychol.* 78 753–762. 10.1037/0021-9010.78.5.753

[B23] Janssen O. Van de Vliert E. West M. (2004). The bright and dark sides of individual and group innovation: a special issue introduction. *J. Organ. Behav.* 25 129–145. 10.1002/job.242

[B24] Ji Y. Yoon H. J. (2021). The effect of servant leadership on self-efficacy and innovative behaviour: verification of the moderated mediating effect of vocational calling. *Admin. Sci.* 11:39. 10.3390/admsci11020039

[B25] Karanika-Murray M. Michaelides G. Wood S. J. (2017). Job demands, job control, psychological climate, and job satisfaction. *J. Organ. Effect.* 4 238–255. 10.1108/joepp-02-2017-0012

[B26] Lee W. R. Choi S. B. Kang S.-W. (2021). How leaders’ positive feedback influences employees’ innovative behavior: the mediating role of voice behavior and job autonomy. *Sustainability* 13:1901. 10.3390/su13041901

[B27] Malik M. A. R. Butt A. N. Choi J. N. (2015). Rewards and employee creative performance: moderating effects of creative self-efficacy, reward importance, and locus of control. *J. Organ. Behav.* 36 59–74. 10.1002/job.1943

[B28] Martín-Hernández P. Ramos J. Zornoza A. Lira E. M. Peiró J. M. (2020). Mindfulness and job control as moderators of the relationship between demands and innovative work behaviours. *J. Work Organ. Psychol.* 36 95–101. 10.1037/ocp0000102 29283600

[B29] Michael L. H. Hou S. T. Fan H. L. (2011). Creative self-efficacy and innovative behavior in a service setting: optimism as a moderator. *J. Creat. Behav.* 45 258–272. 10.1002/j.2162-6057.2011.tb01430.x

[B30] Montani F. Vandenberghe C. Khedhaouria A. Courcy F. (2020). Examining the inverted U-shaped relationship between workload and innovative work behavior: the role of work engagement and mindfulness. *Hum. Relat.* 73 59–93. 10.1177/0018726718819055

[B31] Orth M. Volmer J. (2017). Daily within-person effects of job autonomy and work engagement on innovative behaviour: the cross-level moderating role of creative self-efficacy. *Eur. J. Work Organ. Psychol.* 26 601–612. 10.1080/1359432x.2017.1332042

[B32] Rasool S. F. Samma M. Wang M. Yan Z. Zhang Y. (2019). How human resource management practices translate into sustainable organizational performance: the mediating role of product, process and knowledge innovation. *Psychol. Res. Behav. Manage.* 12 1009–1025. 10.2147/PRBM.S204662 31802958 PMC6830386

[B33] Rasool S. F. Wang M. Tang M. Saeed A. Iqbal J. (2021). How toxic workplace environment effects the employee engagement: the mediating role of organizational support and employee wellbeing. *Int. J. Environ. Res. Public Health* 18:2294. 10.3390/ijerph18052294 33652564 PMC7956351

[B34] Rasool S. F. Wang M. Zhang Y. Samma M. (2020). Sustainable work performance: the roles of workplace violence and occupational stress. *Int. J. Environ. Res. Public Health* 17:912. 10.3390/ijerph17030912 32024195 PMC7037902

[B35] Ryan R. M. Deci E. L. (2000b). Self-determination theory and the facilitation of intrinsic motivation, social development, and well-being. *Am. Psychol.* 55 68–78. 10.1037//0003-066x.55.1.68 11392867

[B36] Ryan R. M. Deci E. L. (2000a). Intrinsic and extrinsic motivations: classic definitions and new directions. *Contemp. Educ. Psychol.* 25 54–67. 10.1006/ceps.1999.1020 10620381

[B37] Ryan R. M. Deci E. L. (2017). *Self-Determination Theory: Basic Psychological Needs in Motivation, Development, and Wellness.* New York, NY: Guilford Publications.

[B38] Ryan R. M. Deci E. L. (2020). Intrinsic and extrinsic motivation from a self-determination theory perspective: definitions, theory, practices, and future directions. *Contemp. Educ. Psychol.* 61:101860. 10.1016/j.cedpsych.2020.101860

[B39] Schermuly C. C. Meyer B. Dämmer L. (2013). Leader-member exchange and innovative behavior: the mediating role of psychological empowerment. *J. Pers. Psychol.* 12:132. 10.1027/1866-5888/a000093

[B40] Schultz P. P. Ryan R. M. Niemiec C. P. Legate N. Williams G. C. (2014). Mindfulness, work climate, and psychological need satisfaction in employee well-being. *Mindfulness* 6 971–985. 10.1007/s12671-014-0338-7

[B41] Scott S. G. Bruce R. A. (1994). Determinants of innovative behavior: a path model of individual innovation in the workplace. *Acad. Manage. J.* 37 580–607. 10.5465/256701

[B42] Sheldon K. M. Prentice M. (2019). Self-determination theory as a foundation for personality researchers. *J. Pers.* 87 5–14. 10.1111/jopy.12360 29144550

[B43] Singh M. Sarkar A. (2012). The relationship between psychological empowerment and innovative behavior. *J. Pers. Psychol.* 11 127–137. 10.1027/1866-5888/a000065

[B44] Singh M. Sarkar A. (2019). Role of psychological empowerment in the relationship between structural empowerment and innovative behavior. *Manage. Res. Rev.* 42 521–538. 10.1108/mrr-04-2018-0158

[B45] Slåtten T. Mutonyi B. R. Lien G. (2020). The impact of individual creativity, psychological capital, and leadership autonomy support on hospital employees’ innovative behaviour. *BMC Health Serv. Res.* 20:1096. 10.1186/s12913-020-05954-4 33246454 PMC7691957

[B46] Stashevsky S. Burke R. Carmeli A. Meitar R. Weisberg J. (2006). Self-leadership skills and innovative behavior at work. *Int. J. Manpower* 27 75–90. 10.1108/01437720610652853

[B47] Su W. Lin X. Ding H. (2019). The influence of supervisor developmental feedback on employee innovative behavior: a moderated mediation model. *Front. Psychol.* 10:1581. 10.3389/fpsyg.2019.01581 31338055 PMC6629885

[B48] Su W. Lyu B. Chen H. Zhang Y. (2020). How does servant leadership influence employees’ service innovative behavior? The roles of intrinsic motivation and identification with the leader. *Baltic J. Manage.* 15 571–586. 10.1108/bjm-09-2019-0335

[B49] Sweet S. N. Fortier M. S. Strachan S. M. Blanchard C. M. (2012). Testing and integrating self-determination theory and self-efficacy theory in a physical activity context. *Can. Psychol.* 53 319–327. 10.1037/a0030280

[B50] Sweet S. N. Fortier M. S. Strachan S. M. Blanchard C. M. Boulay P. (2014). Testing a longitudinal integrated self-efficacy and self-determination theory model for physical activity post-cardiac rehabilitation. *Health Psychol. Res.* 2:1008. 10.4081/hpr.2014.1008 26973926 PMC4768554

[B51] Tierney P. Farmer S. M. (2002). Creative self-efficacy: its potential antecedents and relationship to creative performance. *Acad. Manage. J.* 45 1137–1148. 10.5465/3069429

[B52] Yidong T. Xinxin L. (2013). How ethical leadership influence employees’ innovative work behavior: a perspective of intrinsic motivation. *J. Bus. Ethics* 116 441–455. 10.1007/s10551-012-1455-7

[B53] Zhang X. Bartol K. M. (2010). Linking empowering leadership and employee creativity: the influence of psychological empowerment, intrinsic motivation, and creative process engagement. *Acad. Manage. J.* 53 107–128. 10.5465/amj.2010.48037118

